# Suicidal ideation and non-fatal suicidal self-directed violence prevalence and associations among Veterans residing in U.S. Pacific Island Territories: Guam, American Samoa, and the Northern Mariana Islands

**DOI:** 10.1371/journal.pone.0326533

**Published:** 2025-06-18

**Authors:** Lindsey L. Monteith, Julie A. Kittel, Ryan Holliday, Alexandra L. Schneider, Evan Herring-Nathan, Lauren S. Krishnamurti, Lisa A. Brenner, Claire A. Hoffmire

**Affiliations:** 1 Rocky Mountain Mental Illness Research, Education and Clinical Center for Suicide Prevention, Department of Veterans Affairs, Aurora, Colorado, United States of America; 2 Spark M. Matsunaga VA Medical Center, VA Pacific Islands Healthcare System, Honolulu, Hawaii, United States of America; 3 Department of Physical Medicine and Rehabilitation, University of Colorado Anschutz Medical Campus, Aurora, Colorado, United States of America; 4 Department of Psychiatry, University of Colorado Anschutz Medical Campus, Aurora, Colorado, United States of America; 5 Firearm Injury Prevention Initiative, University of Colorado Anschutz School of Medicine, Aurora, Colorado, United States of America; 6 NORC at the University of Chicago, Chicago, Illinois, United States of America; The Quinism Foundation, UNITED STATES OF AMERICA

## Abstract

Suicide rates are high in United States (U.S.) Pacific Island Territories, where large numbers of Veterans reside. Yet knowledge of suicidal self-directed violence (SSDV) among Veterans in this region is limited. We examined the feasibility of surveying Veterans in Guam, the Commonwealth of the Northern Mariana Islands (CNMI), and American Samoa regarding suicidal ideation (SI) and non-fatal SSDV, and examined SI and non-fatal SSDV prevalence and associations with SI. Of 3,000 Veterans invited to participate (2022), 566 completed the survey (21.6% response rate). Population-based SI estimates were 35.86% (95% CI: 28.34, 43.39) for lifetime and 15.68% (95% CI: 10.91, 20.44) for past year. The prevalence of post-military and past-year SI was significantly higher among Veterans ages 18–34. SI prevalence was also significantly higher among American Indian/Alaska Native Veterans and was significantly lower among Samoan Veterans. The prevalence of SI and suicide attempts (lifetime and during military service) appeared to be higher among Veterans residing in U.S. Pacific Island Territories, compared to among Veterans in all 50 states, DC, and Puerto Rico; however, these differences were not statistically significant. The most common methods considered during past-year SI were motor vehicle crash (32.77%; 95% CI: 19.38, 46.16), overdose/poisoning (26.12%; 95% CI: 15.02, 37.22), and gunshot (24.30%; 95% CI: 11.98, 36.62). Lifetime prevalence was 11.84% (95% CI: 11.05, 12.62) for preparatory behavior(s), 11.96% (95% CI: 7.88, 16.05) for interrupted attempts, and 9.86% (95% CI: 6.36, 13.37) for suicide attempts. Inclusion of Veterans from Pacific Island Territories in suicide prevention surveillance and research is feasible and vital to inform suicide prevention in this region. Prevention efforts targeted to Veterans ages 18–34 are also warranted.

## Introduction

In 2021, United States (U.S.) military Veterans experienced an age- and sex-adjusted suicide rate 71.8% higher than that of non-Veteran U.S. adults [1]. To address the elevated suicide rate among U.S. Veterans [1], the Department of Veterans Affairs (VA) has made suicide prevention its top clinical priority, implementing an evidence-based public health approach that includes numerous strategies to prevent suicide among *all* Veterans [2]. One key component of this strategy is surveillance. Each year, the VA publishes a National Suicide Prevention Report, which describes rates of suicide in the U.S. Veteran population, how rates have changed over time, and how rates vary between different groups, such as by sex, age, race, ethnicity, and use of Veterans Health Administration (VHA) services [1,3]. In addition to the national report, the VA’s annual state-based reports provide crucial state-based information regarding the number and rate of Veteran suicides in each state, along with comparisons to regional and national rates [4]. The state data sheets are considered “a critical tool to help VA and state-level partners design and execute the most effective suicide prevention strategies” [4]. Annual information from the national and state-specific reports has been instrumental in informing regional suicide prevention efforts.

While crucial information has been available on national and state-level suicide numbers and rates, information has been more limited regarding Veteran suicide in U.S. Pacific Island Territories (i.e., Guam, the Commonwealth of the Northern Mariana Islands [CNMI], and American Samoa), where 10,000 or more Veterans reside [5–7]. Data regarding suicide deaths that occurred in U.S. Pacific Island Territories was not included in National Death Index records until 2017, and even those records “are not considered complete for all years” [8]. Thus, such information historically was not able to be included in national VA suicide prevention reports. Nonetheless, the *VA National Strategy for Preventing Veteran Suicide* endeavors to support efforts by U.S. territories to use Veteran suicide data [2], and there is now a U.S. Territories report [9] and associated information (specific to 2017–2021) in the state/territory data appendix [10]. However, due to the need to protect privacy when there are small numbers of deaths, these include data aggregated from the U.S. Virgin Islands and U.S. Pacific Island Territories, with limited information regarding the number of lives lost to suicide, suicide rates, or changes over time [9,10]. Consequently, information regarding Veteran suicide in U.S. Pacific Island Territories remains limited.

The dearth of such information is concerning not only because it precludes understanding the extent to which suicide is a problem among Veterans in U.S. Pacific Island Territories, but also because general population suicide rates are high in this region. Guam—which has the largest Veteran population of all U.S. Pacific Island Territories, estimated at 10,000 in 2017 [6]—had age-adjusted suicide rates of 30.0 and 21.2 per 100,000 in 2020 and 2021 [11]. These rates are considerably higher than national U.S. suicide rates in 2020 and 2021, which were 13.5 and 14.1 per 100,000, respectively [12–13]. In addition, in the Western Pacific, which includes numerous nations across multiple continents (e.g., Australia, Asia), suicide rates were particularly high in specific Pacific Island nations, such as the Federated States of Micronesia and Kiribati, which had crude rates of 28.2 and 28.3 per 100,000, respectively, in 2019 [14].

In addition to the high suicide rates in Guam and the Pacific Island region [11,14,15], extrapolating findings from the overall Veteran population to Veterans residing in U.S. Pacific Island Territories may be problematic for additional reasons. Asian American, Native Hawaiian, and Pacific Islander (AANHPI) Veterans comprise a numerical minority (3.0%) of the overall Veteran population, with 2.6% of Veterans identifying as Asian alone and 0.4% identifying as Native Hawaiian or Pacific Islander alone [16]; however, AANHPI individuals constitute the majority of the populace of U.S Pacific Island Territories. Specifically, 93.0% of the population of American Samoa [17], 54.2% in Guam [18], and 50.3% in CNMI [19] identify as Pacific Islander or Native Hawaiian. This is relevant as AANHPI Veterans experienced a drastic increase (192.6%) in suicide rates from 2001–2021 (i.e., unadjusted rates of 10.8 and 31.6 per 100,000 in 2001 and 2021, respectively) that far exceeded changes among other racial and ethnic groups of Veterans, which ranged from a decrease of 46.2% among those with unknown race to an increase of 78.8% among Veterans who identified as American Indian/Alaska Native (AI/AN) [3]. In addition, U.S Pacific Island Territories have distinct histories, indigenous cultures, and geographies from the Continental U.S. [20–22], which may contribute to regional differences in rates of suicidal self-directed violence (SSDV; defined as self-directed behavior, with suicidal intent, which deliberately results in actual or potential injury to oneself [23]).

Thus, it is vital to elucidate the prevalence of SSDV among Veterans in U.S Pacific Island Territories. Given challenges ascertaining rates of suicide among Veterans in this region, understanding the prevalence of suicidal ideation (SI) and non-fatal suicidal self-directed violence (NF-SSDV; e.g., suicide attempts) among Veterans in U.S Pacific Island Territories is critical. When systematically assessed over time, prevalence data can also be used to make inferences regarding the potential effectiveness of regional suicide prevention efforts, which are ongoing across U.S. states and territories [24]. To our knowledge, no studies have examined the prevalence of SI or NF-SSDV among Veterans residing in U.S. Pacific Island Territories.

### Aims

Given the absence of prior research on SI and NF-SSDV among Veterans in U.S. Pacific Island Territories, as well as limited knowledge available to determine if surveying Veterans in this region would be feasible (e.g., response rates), we conducted a pilot study of U.S. Veterans residing in this region as part of a larger study (*Assessing Social and Community Environments with National Data* *for Veteran Suicide Prevention* [ASCEND] [25]) to determine the feasibility of surveying Veterans in this region (i.e., response rates) and to estimate NF-SSDV prevalence. In this manuscript, we report on: (1) response and yield rates; (2) SI and NF-SSDV prevalence, including comparing the prevalence of SI and suicide attempts among Veterans in U.S. Pacific Island Territories to that of Veterans nationally (i.e., those residing in all 50 U.S. states, Washington D.C., and Puerto Rico); and (3) associations between demographic and VA characteristics with post-military and past-year SI. We examined prevalence and associations among Veterans in U.S. Pacific Island Territories overall (i.e., those residing in Guam, CNMI, or American Samoa), as well as specifically among Veterans residing in Guam, the Pacific Island Territory with the largest number of Veterans [5–6].

## Materials and methods

ASCEND seeks to determine the prevalence of SI and different types of NF-SSDV, as well as risk and protective factors, in the overall U.S. Veteran population, among specific subgroups (e.g., by gender, race/ethnicity), and by region, through recurring, cross-sectional surveys [25]. In 2020, an initial pilot study, which had a response rate of 17.5%, was conducted to determine optimal methods, including response mode options, incentive amounts, and survey duration and content [26–27]. Results were used to establish the methods employed in the first large-scale wave of ASCEND data collection in 2022 (Wave 1) [28]. However, the 2020 pilot study did not sample or include any Veterans residing in Pacific Island Territories; thus, the present study (i.e., the Wave 1 Pacific Island Territories pilot study) served as a region-specific assessment of the feasibility of data collection within the larger ASCEND initiative.

### Sampling

Wave 1 included two geographically defined samples: the main sample (i.e., Veterans living in all 50 U.S. states, Washington D.C., and Puerto Rico [28]), and the Pacific Island Territories pilot sample, which included Veterans residing in Guam, CNMI, and American Samoa. Both samples were drawn from the population of approximately 16 million U.S. Veterans alive in June 2021. Two data sources were used to construct the final sampling frame (N = 16,738,616 eligible cases): (1) the U.S. Veterans Eligibility Trends and Statistics (USVETS) Database, which provides comprehensive information for all living U.S. Veterans; and (2) the VA-DoD Identity Repository (VADIR), which contained all Veterans whose most recent separation spanned Fiscal Year 2018 (10/01/2017) to the beginning of sampling (1/1/2022), used as a secondary frame source to ensure comprehensive coverage of Veterans who recently separated from military service. For the main sample, data within these sources were used to implement stratified random sampling with respect to state of residence, separation date, sex, and race/ethnicity. Records were excluded for individuals who were: (1) deceased; (2) not located in a qualifying state/territory; (3) missing state of residence; (4) missing personally identifying information (first name, last name, address, city, state, or zip code) necessary for contact updating; or (5) not Veterans. Additional details are available [28].

The Pacific Island Territories pilot study utilized simple random sampling, distributed in proportion to the size of the Veteran population in each Pacific Island Territory. Only information available in the sampling frame could be used to contact potential participants, as the contact locating service used did not cover these territories. From February 24, 2022 through May 16, 2022, 3,000 Veterans were sent invitations to participate in the Pacific Island Territories pilot study. This number was determined based on a desire to obtain at least 150 completed surveys from Veterans in this region to ensure adequate precision for NF-SSDV estimates and assuming a very conservative response rate of 5%, considering the lack of prior studies available to guide expectations regarding response rates from Veterans in this region and due to logistical considerations that could negatively impact response (e.g., inability to update mailing addresses; time zone differences limiting the utility of the telephone response mode).

### Procedures

Wave 1 used a 10-week, sequential, multimode, push-to-web design, which included three data collection modes: online (primary), paper (secondary), and telephone (available only upon request). Veterans were initially sent an invitation by mail and email (if an email address was available) to participate online, along with a $1 pre-incentive. Subsequent invitations included bi-weekly reminder mailings (Weeks 2, 4, 6, and 8), telephone prompting (beginning at Week 4, using telephone numbers from the sampling frame for Veterans in the Pacific Islands pilot), and a mailed paper survey to non-responders (Week 6).

Ethics approval to conduct this study was obtained from the Colorado Multiple Institutional Review Board and the VA Eastern Colorado Healthcare System Research and Development Committee (Protocol 20–0719). Informed consent was obtained from all participants prior to initiating study procedures. To facilitate participants in different locations participating through various modes (i.e., online, mailed survey, or telephone), the IRB approved a waiver of documentation of consent for this study. Thus, for individuals who participated by telephone, consent was obtained verbally. For individuals who participated online, participants conveyed their consent by checking a box to confirm that they had reviewed the consent form, understood the information contained within it, and agreed to participate. For individuals who participated via mailed paper survey, they communicated consent by returning the mailed paper survey to the study team. All potential respondents were informed that they could call a toll-free study help desk if they had any questions or concerns about the study or wanted additional information. Respondents received a $5 post-incentive for their participation, unless they declined to receive the post-incentive.

### Measures

The ASCEND survey was developed through expert consultation, review of the existing literature, and consultation with a project-specific Veterans Engagement Board [29]. Constructs examined in the present analyses are described below.

#### SI and NF-SSDV.

We administered the *ASCEND Non-Fatal Suicidal Self-Directed Violence Module*. This module included questions adapted from the Columbia-Suicide Severity Rating Scale (C-SSRS [30]), including the Army STARRS adaptation [31], as well as questions from the Self-Injurious Thoughts and Behaviors Interview (SITBI [32)]. Items also assessed lifetime preparatory behaviors [28]. Thus, the NF-SSDV module assessed lifetime SI experiences (i.e., thoughts of killing oneself), with and without consideration of method and a specific plan; interrupted attempts (i.e., started to attempt suicide, but stopped self or was stopped by someone or something prior to beginning to hurt oneself); and suicide attempts (i.e., purposefully hurt self with at least some intent to die). For respondents who reported SI, interrupted attempts, and/or suicide attempts, additional follow-up questions were asked regarding timing relative to military service (i.e., preceding, during, and following). Among those who reported lifetime SI, additional questions assessed SI during more recent periods (i.e., past year, past month), methods considered during past-year SI, and preparatory behaviors (e.g., researching ways to kill oneself, getting affairs in order). Among respondents who reported lifetime suicide attempt(s), additional information was assessed, such as injury severity (i.e., most severe), methods used, and substance use (i.e., alcohol, drugs, or non-prescribed medications) immediately before or during any suicide attempts.

#### Potential SI correlates.

Constructs of interest for examining associations with SI included age, region of residence, race/ethnicity, if multi-racial, gender, VA service connection, VHA enrollment, and use of VHA services. All of these except for region were based on self-report. Age was categorized into four groups (18–34; 35–54; 55–74; 75+) to mirror the age categories within the annual VA Suicide Prevention Reports [1]. Region of residence was categorized into three groups (Guam, CNMI, or American Samoa), determined via initial sampling frame contact information, unless the location provided was different, in which case the more current location was used. For race, respondents could select multiple response options from the following: White, Black or African American, AI/AN, Asian Indian, Chinese, Filipino, Japanese, Korean, Vietnamese, Other Asian, Native Hawaiian, Guamanian or CHamoru, Samoan, Other Pacific Islander, and some other race; some of these were collapsed during analysis due to small n’s (<10; described in more detail below). Additionally, those who selected “Other Asian” or “some other race” were prompted to provide additional information, and their free text responses were reviewed; if their free text response could be categorized into an existing response category, it was re-categorized to reflect Census categories. Ethnicity was also assessed via self-report and categorized as Hispanic or non-Hispanic. Multi-racial was operationalized as anyone who endorsed more than one race. Gender was categorized into men and women, as no respondents selected a different response option. VA service connection (i.e., if they reported having a VA service-connected disability rating; yes/no), VHA enrollment (i.e., if they had ever enrolled in VA healthcare; yes/no), and use of services provided by VHA (never, within the year preceding the survey, or more than a year ago) were also assessed via self-report.

#### Descriptives.

To describe the sample, the aforementioned variables were examined, along with additional demographic and military service characteristics that were also assessed via self-report. These included sexual orientation, marital/relationship status, caregiver for minors or adults, education, employment, service era, branch of active-duty service, and deployment history.

### Analysis

All estimates of survey data reporting SI and NF-SSDV prevalence and examining associations with SI were weighted to enhance generalizability to all Veterans residing in Pacific Island Territories. Analyses were conducted using SUDAAN version 11.0.3, SAS version 9.4, and R version 4.3.2. When R was used to provide weighted estimates, the *survey* package [33] was used. For results that included weighted prevalence estimates, unweighted frequencies are reported, followed by weighted frequencies and percentages corresponding to population-based estimates, with 95% Confidence Intervals (CIs).

#### Weighting procedure.

The final weights used in all analyses were developed to adjust for known eligibility and differential non-response, and to ensure that the final weighted sample was aligned with the total number (N = 9,517) and demographic composition of all Veterans residing in the Pacific Island Territories. The non-response adjustment was informed by an analysis of the full ASCEND sample (i.e., inclusive of the main sample and the Pacific Island Territories pilot sample), which revealed that race/ethnicity, rurality, VHA use, age, and recency of separation were associated with response propensity [28]. Veterans of color, in rural locations, and who had not recently used VHA care had lower response rates; additionally, response propensity was positively correlated with age and time since separation (i.e., increased with increasing age and time since separation). The non-response adjustment was used to account for this differential nonresponse among sampled cases with known eligibility. Raking was used to ensure that final survey weights aligned with known characteristics of the Veteran population (i.e., to compensate for any residual undercoverage). Raking benchmarks were initially tabulated from the study sampling frame (i.e., USVETS and VADIR) and were specific to the Pacific Island Territories, as we identified from the frame that the demographic composition of Veterans residing in Pacific Island Territories differed from that of Veterans in the main sample. The initial counts were then adjusted based on observed reported eligibility rates from the screener-responding cases. The resulting benchmarks represent our best estimate of the number of Veterans residing in these territories. Weights were constructed using SAS version 9.4.

#### Survey response (Aim 1).

We examined response rates, computed as completion yield (total completes divided by the total sample; the most conservative approach to estimating response rates as it does not include the subtraction of ineligible and not contacted Veterans from the denominator), as well as rates based on American Association for Public Opinion Research (AAPOR) standard definitions [34]. Response rate calculations included all participants who completed the survey (n = 566), including 13 participants who reported when they provided their address to receive their post-incentive payment that they no longer lived in a U.S. Pacific Island Territory. Because examining response rates by subgroup requires demographic information about individuals who completed and did not complete the survey, all subgroup variables for Aim 1 were sourced from the sampling frame (i.e., USVETS and VADIR), rather than from self-reported survey data. Building upon the broader non-response analysis, we conducted chi-square tests (unweighted) to examine if yield rates specific to Pacific Island Territories significantly differed by sampling frame variables, including race/ethnicity, sex, age, region of residence, frame source, recency of separation, and VHA use.

#### Prevalence of SI and NF-SSDV (Aim 2).

The 13 participants who did not reside in a U.S. Pacific Island territory at the time of participation were excluded from further analyses, resulting in an analytic sample of n = 553 for analyses examining prevalence and associations. To examine NF-SSDV prevalence, weighted population-based estimates were calculated, along with 95% CIs, both for Veterans residing in all U.S. Pacific Island Territories, as well as specifically for Veterans residing in Guam. Prevalence was not calculated separately for Veterans residing in CNMI and American Samoa due to lower numbers of participants from these territories. Prevalence estimates were only reported if they met the multi-step National Center for Health Statistics (NCHS) Data Reporting Standards for Proportions [35]. Comparisons were made between Veterans residing in U.S. Pacific Island Territories and Veterans in the ASCEND main sample (previously computed [28]) by examining 95% CIs; if the CIs did not overlap, differences were considered significant.

#### Associations with SI (Aim 3).

Weighted prevalence ratio (PR) estimates and 95% CIs for all correlates of interest were computed for post-military SI and past-year SI. Weighted PR estimates were directly computed via Poisson regression models with robust standard errors via the *survey* package in R. For binary variables, reference groups were selected based on being the most populous group, a natural reference group, or based on prior research (e.g., for age), with the exception of race/ethnicity. For race/ethnicity, due to lack of mutual exclusivity between variables, the PR was computed for each race/ethnicity relative to all other races/ethnicities, rather than using a constant reference group. This was done for all specific racial/ethnic groups that had *n*’s ≥10; consequently, these analyses could not be computed for some racial/ethnic groups, such as Native Hawaiian, or “some other race,” due to small sample sizes); specific Asian ethnicities (Asian Indian, Chinese, Korean, Vietnamese, and Other Asian) were also collapsed into a broader category due to small *n*’s. For all models, results based upon cells with *n* < 10 were not conducted (e.g., there were zero participants who identified as Samoan who resided in Guam who reported past-year SI).

## Results

### Response rates (Aim 1)

The ASCEND Wave 1 Pacific Island Territories pilot study yielded 566 completed surveys, which corresponded to a yield rate of 18.9% and an AAPOR response rate of 21.6%. As per design, web was the most common response mode (75.6% of respondents), whereas paper (24.2%) and telephone (0.2%) were less common. Yield rates significantly differed across all sampling frame variables examined (Table 1), including those identified in the broader study non-response analysis [28] and incorporated into design weights (state/territory of residence, sex, race/ethnicity, and recency of separation). Specifically, yield was lowest among Veterans whose race/ethnicity was listed as non-Hispanic AANHPI (15.97%), followed by non-Hispanic White (20.77%), Hispanic (23.76%), non-Hispanic Black (24.39%), and non-Hispanic Other (29.40%) Veterans. In addition, no one in the sample frame with unknown race/ethnicity responded to the survey, which may be indicative, in part, of missingness in the administrative frame data serving as an indicator of data quality in general (i.e., contact information may be less accurate in these cases). Females (15.09%) had a lower yield rate than males and individuals whose sex was unknown (19.49%). Yield was lowest for Veterans ages 18–34 (12.73%), followed by those ages 35–49 (17.62%), ≥ 65 (17.98%), and 50–64 (22.62%). Comparing yield by region, Veterans in American Samoa had the lowest yield rate (14.35%), followed by those in Guam (18.80%); Veterans in CNMI had the highest yield rate (25.57%). Veterans sourced from VADIR (15.34%) had lower yield than those sourced from USVETS (19.60%). Closely related to age and frame source, yield was lowest among those who separated ≤5 years ago (13.96%) and higher among those who separated between 6–10 years ago (19.10%) and ≥11 years ago (19.91%). Yield was lower among Veterans who had not used any VHA services in the Fiscal Year prior to USVETS frame data compilation (i.e., Fiscal Year 2019; 16.25%), compared to those who had used such services in Fiscal Year 2019 (26.35%).

**Table 1:** Completion yield rates among Veterans in U.S. Pacific Island Territories (N = 3,000).

	Complete (n)	Not Complete (n)	Yield Rate (%)	Chi-square
**Overall**	566	2,434	18.87	
**Race/ethnicity**				Χ^2^(5)=89.4, *p* < .001
Unknown	0	179	0	
Non-Hispanic AANHPI	236	1,242	15.97	
Non-Hispanic White	97	370	20.77	
Hispanic	86	276	23.76	
Non-Hispanic Black	20	62	24.39	
Non-Hispanic Other	127	305	29.40	
**Sex**				Χ^2^(1)=4.3, *p* = .038
Female	64	360	15.09	
Male or unknown	502	2,074	19.49	
**Age (years)**				Χ^2^(3)=16.3, *p* < .001
18-34	34	233	12.73	
35-49	108	505	17.62	
50-64	209	715	22.62	
≥65	215	981	17.98	
**Region of residence**				
American Samoa	32	191	14.35	Χ^2^(2)=8.1, *p = .*017
Guam	489	2,112	18.80	
Northern Mariana Islands	45	131	25.57	
**Sampling frame source**				Χ^2^(1)=4.8, *p = *.029
VADIR	79	436	15.34	
USVETS	487	1,998	19.60	
**Recency of separation**				Χ^2^(2)=9.2 *p* = .010
≤5 years	68	419	13.96	
6-10 years	55	233	19.10	
≥11 years	443	1,782	19.91	
**VHA use (FY 2019)**				Χ^2^(1)=37.8, *p* < .001
No	361	1,861	16.25	
Yes	205	573	26.35	

All of these subgroup variables were sourced from the sampling frame. AANHPI = Asian American, Native Hawaiian, or Pacific Islander; FY = Fiscal Year; USVETS = United States Veterans Eligibility Trends and Statistics; VADIR = VA-DoD Identity Repository; VHA = Veterans Health Administration.

### Sample characteristics

Weighted sample characteristics are included in Table 2 for the overall population of Veterans residing in all U.S. Pacific Island Territories and specifically for Veterans residing in Guam. The largest percentages of respondents were in the 55–74 age group, followed by 35–54, then 18–34, with only a small proportion 75 and older. The majority of Veterans residing in U.S. Pacific Island Territories resided in Guam, with smaller percentages in CNMI and American Samoa. Nearly two-thirds of Veterans (65.9%; 95% CI: 58.99, 72.90) identified as Pacific Islander, which was the most common overarching racial category. When disaggregated, Guamanian or CHamoru was most common (nearly half), followed by White (nearly one-third), then Black or African American, Hispanic/Latinx, Samoan, Filipino, Other Asian, and Other Pacific Islander; AI/AN, Japanese, Native Hawaiian, and some other race each comprised relatively small percentages of the population. Approximately one-quarter (24.0%; 95% CI: 10.83, 37.12) identified as multi-racial. The majority were men, heterosexual, and currently married. Over one-third currently had one or more minors in their care; a similar proportion were currently caring for other adults. Education varied widely, with the largest proportion having completed some college. Over half were currently employed, and over one-quarter were retired. Service era and military branch varied, with more recent service eras most common and Army the most common branch (over half). The vast majority had deployed. Nearly half were VA service-connected. Over half had enrolled in VHA care; however, only 10.3% (95% CI: 6.97, 13.70) had used VHA services in the past year, and 51.6% (95% CI: 42.31, 60.96) indicated that they had never used any VHA services.

**Table 2:** Unweighted Frequencies and Weighted Sample Characteristics.

	All Pacific Island Territories (n = 553)					Guam (n = 485)				
	**n**	**Weighted n**	**%**	**95% CI**		**n**	**Weighted n**	**%**	**95% CI**	
**Age** ^**a**^										
18-34	29	1,500	16.05	10.28	21.82	21	1,100	13.54	7.73	19.35
35-54	157	3,057	32.71	25.84	39.58	135	2,632	32.38	24.78	39.98
55-74	288	4,272	45.72	35.78	55.66	259	3,928	48.32	37.49	59.16
75+	65	515	5.52	3.87	7.16	59	468	5.76	3.90	7.62
**Region of residence**										
Guam	485	8,264	86.84	82.73	90.94					
Northern Mariana Islands	41	817	8.58	5.25	11.92					
American Samoa	27	436	4.58	2.38	6.78					
**Race/ethnicity** ^**b,c**^										
Guamanian or CHamoru	285	4,441	47.27	38.45	56.08	260	3,877	47.48	37.54	57.42
White	123	2,898	30.85	19.07	42.62	115	2,764	33.86	20.88	46.84
Black or African American	26	1,250	13.30	0.00	27.63	26	1,250	15.30	0.00	31.42
Hispanic/Latinx	84	1,212	13.23	9.37	17.09	76	1,059	13.32	9.20	17.46
Samoan	30	1,210	12.88	0.00	27.32	7	837	10.25	0.00	27.20
Filipino	64	1,130	12.02	8.18	15.86	62	1,047	12.82	8.63	17.02
Other Asian^d^	26	1,097	11.52	0.00	25.92	24	1,083	13.10	0.00	29.39
Other Pacific Islander	49	887	9.44	6.17	12.71	37	626	7.67	4.72	10.61
American Indian or Alaska Native	12	268	2.86	0.84	4.87	11	242	2.96	0.72	5.20
Japanese	11	190	2.02	0.68	3.37	9	146	1.79	0.53	3.04
Native Hawaiian	4	48	0.51	0.00	1.06	4	48	0.59	0.00	1.22
Some other race	4	66	0.70	0.00	1.41	4	66	0.80	0.00	1.63
**Multi-racial**										
No	455	7,055	76.02	62.88	89.17	398	6,048	75.00	60.19	89.82
Yes	80	2,225	23.98	10.83	37.12	72	2,016	25.00	10.18	39.82
**Gender**										
Man	483	8,130	86.76	82.75	90.78	429	7,171	87.94	83.83	90.05
Woman	58	1,240	13.24	9.22	17.25	47	984	12.06	7.95	16.17
**Sexual orientation** ^**e**^										
Heterosexual	482	8,528	93.54	90.67	96.40	424	7,450	94.39	91.65	97.14
Lesbian, gay, bisexual, questioning, or other	32	589	6.46	3.60	9.33	26	443	5.61	2.86	8.35
**Marital/relationship status**										
Married	398	6,811	72.69	66.35	79.03	348	6,011	73.92	67.18	80.66
Never married	40	922	9.84	6.13	13.56	36	793	9.75	5.76	13.74
Widowed	28	281	3.00	1.72	4.28	24	225	2.76	1.48	4.05
Divorced/separated	73	1,355	14.47	10.13	18.80	65	1,103	13.56	9.18	17.94
**Caretaking of minors**										
No	324	5,005	64.33	58.92	69.74	292	4,509	68.20	62.61	73.79
Yes	134	2,775	35.67	30.26	41.08	106	2,102	31.80	26.21	37.39
**Caretaking of adults**										
No	313	5,121	63.34	58.46	69.74	281	4,450	64.54	59.42	69.65
Yes	189	2,964	36.67	21.79	41.54	159	2,445	35.46	30.35	40.58
**Education**										
Less than 9th grade or 9th-12th grade, no diploma	9	97	1.04	0.25	1.82	9	97	1.19	0.29	2.10
High school diploma or equivalent	112	1,631	17.38	12.87	21.88	103	1,435	17.62	12.72	22.53
Some college, no college degree	190	3,143	33.49	26.41	40.56	164	2,618	32.14	24.50	39.78
Associate’s degree	85	1,527	16.27	11.66	20.88	74	1,354	16.62	11.45	21.80
Bachelor’s degree	88	1,206	12.85	9.37	16.34	76	1,024	12.57	8.81	16.32
Master’s, doctoral, or professional degree	57	1,781	18.98	5.36	32.60	49	1,618	19.86	4.40	35.31
**Employment**										
Employed	269	5,074	54.37	44.32	64.43	231	4,280	52.83	41.71	63.95
Not employed, retired	182	2,660	28.50	16.25	40.76	166	2,495	30.80	17.14	44.45
Not employed, disabled	40	591	6.33	3.74	8.92	32	425	5.24	2.90	7.59
Not employed, seeking or temporarily laid off	15	267	2.86	1.22	4.50	14	248	3.07	1.23	4.91
Not employed, other	29	740	7.93	4.30	11.56	27	653	8.06	4.12	12.00
**Service era** ^**b**^										
July 1964 or prior	27	222	2.33	1.36	3.30	23	188	2.28	1.24	3.31
August 1964 to April 1975 (Vietnam era)	159	1,289	13.74	10.56	16.91	144	1,179	14.50	10.81	18.18
May 1975 to July 1990	296	4,484	47.77	38.14	57.41	259	4,053	49.83	39.22	60.43
August 1990 to August 2001 (includes Gulf War)	222	3,275	34.89	27.80	41.99	196	2,898	35.62	27.54	43.71
September 2001 or later	204	4,557	48.55	39.39	57.71	176	3,793	46.63	36.57	56.69
**Branch**^**b**^ **(active-duty)**										
Army	301	4,820	50.65	41.43	59.87	259	4,006	48.47	38.39	58.55
Air Force	119	2,589	27.21	14.91	39.50	112	2,507	30.34	16.78	43.90
Navy	112	1,759	18.48	13.85	23.11	101	1,560	18.88	13.76	24.00
Marine Corps	37	528	5.55	3.21	7.89	30	398	4.82	2.73	6.92
Coast Guard	11	186	1.96	0.68	3.23	8	137	1.66	0.40	2.93
**Deployments**										
No	104	1,867	20.42	15.22	25.61	84	1,468	18.55	13.16	23.93
Yes	417	7,279	79.58	74.39	84.78	371	6,446	81.45	76.07	86.84
**VA service connection**										
No	253	5,017	53.26	44.34	62.18	229	4,504	55.01	45.27	64.74
Yes	291	4,403	46.74	37.82	55.66	249	3,684	45.00	35.26	54.73
**Enrolled in VHA**										
No	177	3,725	43.37	31.34	53.40	161	3,380	44.65	32.45	56.85
Yes	338	5,068	57.63	46.60	68.66	288	4,190	55.35	43.15	67.55
**Use of VHA services**										
No	221	4,739	51.63	42.31	60.96	203	4,309	54.12	44.04	64.12
Yes, prior to past year	246	3,461	38.03	30.25	45.81	203	2,795	35.11	26.93	43.28
Yes, in the past year	65	948	10.33	6.97	13.70	60	858	10.77	7.06	14.49

Percentages for Veterans in the overall sample were weighted to Veterans residing in U.S. Pacific Island Territories. Percentages for Guam were also weighted.

CI = confidence interval; VHA = Veterans Health Administration.

^a^ Overall: M=53.10, SE=1.26, 95% CI: 50.62, 55.58.

^b^ Not mutually exclu*s*ive.

^c^ Pacific Islander (i.e., endorsed Guamanian or CHamoru, Samoan, or Other Pacific Islander [not mutually exclusive]): Overall: n = 346; weighted n = 6,195; weighted % = 65.94% (95% CI: 58.99, 72.90); Guam: n = 290; weighted n = 5,137; 62.92% (95% CI = 54.57, 71.26). Asian (i.e., Asian Indian, Chinese, Filipino, Japanese, Korean, Vietnamese, Other Asian [not mutually exclusive]): Overall: n = 96; weighted n = 2,328; 24.78% (95% CI: 11.91, 37.65); Guam: n = 91; weighted n = 2,194; 26.88% (95% CI: 12.55, 41.21). For Native Hawaiian, see table.

^d^ Included Korean (n = 7; 1.11% [weighted]), Asian Indian (n = 4; 8.87%), Vietnamese (n = 2; 0.42%), Chinese (n = 2; 0.33%), and Other Asian (n = 13, 1.27%) [not mutually exclusive]. These were included as part of a broader category due to small n’s.

^e^ Due to small numbers of disaggregated categories of sexual orientation, this variable was dichotomized into heterosexual or lesbian, gay, bisexual, questioning, or other.

### Prevalence of SI (Aim 2)

#### Lifetime, by severity.

**Fig 1:**
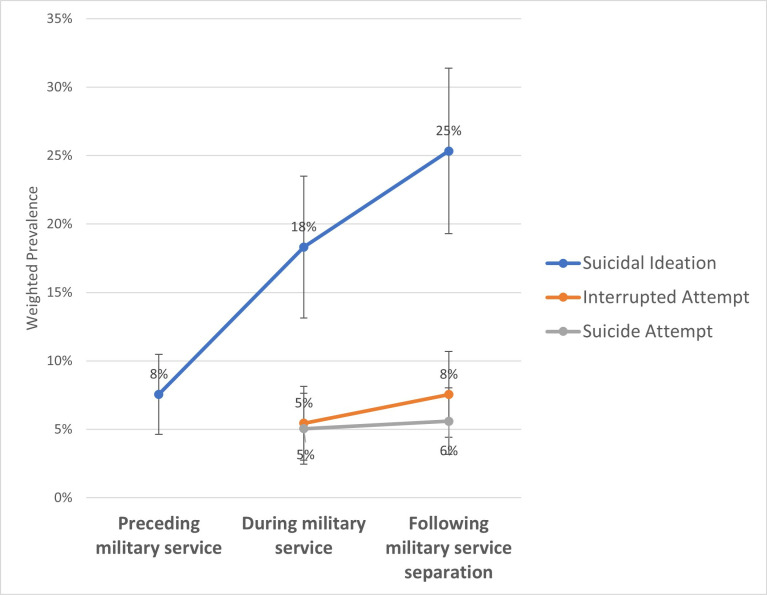
Suicidal Ideation and Non-Fatal Suicidal Self-Directed Violence Timing Relative to Military Service among Veterans Residing in **U.S. Pacific Island Territories.** Values are not displayed for pre-military interrupted attempts and pre-military suicide attempts due to potentially unreliable estimates.

Table 3 displays the weighted prevalence of different severities of lifetime SI and at different periods. Lifetime SI prevalence was 35.86% (95% CI: 28.34, 43.39) for any thoughts of killing oneself, 29.77% (95% CI: 23.12, 36.43) for SI with method considered, and 13.06% (95% CI: 8.86, 17.25) for SI with a specific plan. When examining prevalence relative to military service (Fig 1), 7.56% (95% CI: 4.64, 10.47) experienced SI *prior* to their military service, 18.32% (95% CI: 13.14, 23.50) *during* their service, and 25.34% (95% CI: 19.29, 31.39) at some point *following* separation from military service. SI prevalence in more recent periods included 15.68% (95% CI: 10.91, 20.44) of Veterans in the past year and 5.91% (95% CI: 3.29, 8.53) in the past month.

**Table 3:** Prevalence of lifetime suicidal ideation of varying severities and at different periods.

	All Pacific Island Territories (n = 553)				Guam (n = 485)			
**n**	**%**	**95% CI**		**n**	**%**	**95% CI**	
**Lifetime**								
Thoughts of killing self^a^	172	35.86	28.34	43.39	146	34.85	26.61	43.09
SI with method considered	146	29.77	23.12	36.43	122	28.64	21.43	35.86
SI with a specific plan	63	13.06	8.86	17.25	51	12.08	7.72	16.44
**Relative to Military Service**								
Before	33	7.56	4.64	10.47	28	7.03	4.04	10.02
During	76	18.32	13.14	23.50	65	18.93	13.07	24.78
Following	126	25.34	19.29	31.39	109	24.75	18.19	31.31
**Past Year/Month**								
Past year	71	15.68	10.91	20.44	57	14.88	9.81	19.96
Past month	32	5.91	3.29	8.53	24	5.03	2.55	7.51

CI = confidence interval; SI = suicidal ideation. Frequencies (n’s) presented are unweighted, but all percentages and CIs reflect weighted estimates. All estimates met National Center for Health Statistics Data Presentation Standards for Proportions.

^a^ Irrespective of method or plan.

### Methods

Table 4 reports the prevalence of suicide methods considered during past-year thoughts among Veterans who experienced past-year SI. Motor vehicle crash (32.77%; 95% CI: 19.38, 46.16), overdose or poisoning (26.12%; 95% CI: 15.02, 37.22), and gunshot (24.30%; 95% CI: 11.98, 36.62) were the most prevalent methods considered, followed by jumping from a high place (18.21%; 95% CI: 8.30, 28.12). Hanging, drowning, cutting/stabbing, suffocation/asphyxiation, and other methods were less common (values not reported due to potentially unreliable estimates).

**Table 4:** Suicide methods considered among veterans who experienced past-year suicidal ideation.

	All Pacific Island Territories (n = 71)				Guam (n = 57)			
	n	%	95% CI		n	%	95% CI	
Motor vehicle crash	22	32.77	19.38	46.16				
Overdose or poisoning	24	26.12	15.02	37.22	19	26.79	14.20	39.37
Gunshot	20	24.30	11.98	36.62				
Jumping from a high place	15	18.21	8.30	28.12				

CI = confidence interval. Frequencies (n’s) presented are unweighted, but percentages and CIs reflect weighted estimates. Values not displayed for hanging, drowning, cutting/stabbing, suffocation/asphyxiation, and other methods, due to unreliable estimates. Blank cells reflect suppression in the Guam subsample due to potentially unreliable estimates.

### Prevalence of NF-SSDV (Aim 2)

#### Preparatory behaviors.

Approximately 11.84% (95% CI: 11.05, 12.62) of Veterans in U.S. Pacific Island Territories had engaged in lifetime preparatory behavior(s) (Table 5).

**Table 5:** Prevalence of non-fatal suicidal self-directed violence, by type and period.

	All Pacific Island Territories (n = 553)				Guam (n = 485)			
	n	%	95% CI		n	%	95% CI	
**Preparatory Behaviors**								
Lifetime	60	11.84	11.05	12.62	52	14.04	9.17	18.91
**Interrupted Attempt(s)**								
Lifetime	53	11.96	7.88	16.05	42	10.86	6.66	15.07
During military service	24	5.45	2.75	8.15	17	4.88	2.03	7.73
Following military service	38	7.55	4.41	10.68	32	6.89	3.77	10.01
**Suicide Attempt(s)**								
Lifetime	50	9.86	6.36	13.37	38	8.19	4.84	11.54
During military service	22	5.05	2.46	7.64	17	4.65	1.91	7.39
Following military service	31	5.60	3.17	8.03	23	4.32	2.30	6.34

CI = confidence interval. Frequencies (n’s) presented are unweighted, but percentages and CIs reflect weighted estimates. Values are not displayed for pre-military interrupted attempts, pre-military suicide attempts, and past-year suicide attempts due to unreliable estimates.

#### Interrupted attempts.

Approximately 11.96% (95% CI: 7.88, 16.05) of Veterans had experienced an interrupted suicide attempt in their lifetime (Table 5). Similar to findings regarding SI, the prevalence of interrupted attempt(s) appeared to be higher *following separation* from military service (7.55%; 95% CI: 4.41, 10.68), relative to *during* military service (5.45%; 95% CI: 2.75, 8.15) (Fig 1).

#### Suicide attempts and associated characteristics.

Approximately 9.86% (95% CI: 6.36, 13.37) of Veterans experienced lifetime suicide attempt(s) (Table 5). The prevalence of suicide attempt(s) *during* military service (5.05%; 95% CI: 2.46, 7.64) and *following separation* from military service (5.60%; 95% CI: 3.17, 8.03) were relatively similar (Fig 1).

Respondents with a lifetime history of suicide attempt(s) reported associated injuries. The most common injury severities from suicide attempt(s) were none (35.59%; 95% CI: 19.55, 51.62) and very minor (30.12%; 95% CI: 14.34, 45.90). Minor, moderate or severe injuries were also reported by some participants; however, specific percentages are not reported due to potentially unreliable estimates.

### Prevalence of SI and suicide attempt in U.S. Pacific Island Territories, relative to Veterans overall (Aim 2)

To contextualize how the prevalence of SI and NF-SSDV among Veterans living in U.S. Pacific Island Territories compared to prevalence in the broader U.S. Veteran population, we compared prevalence estimates obtained in the current analyses to those obtained in a prior analysis of the ASCEND Wave 1 main sample (i.e., Veterans living in all 50 U.S. states, Washington D.C., and Puerto Rico [28]).

**Fig 2:**
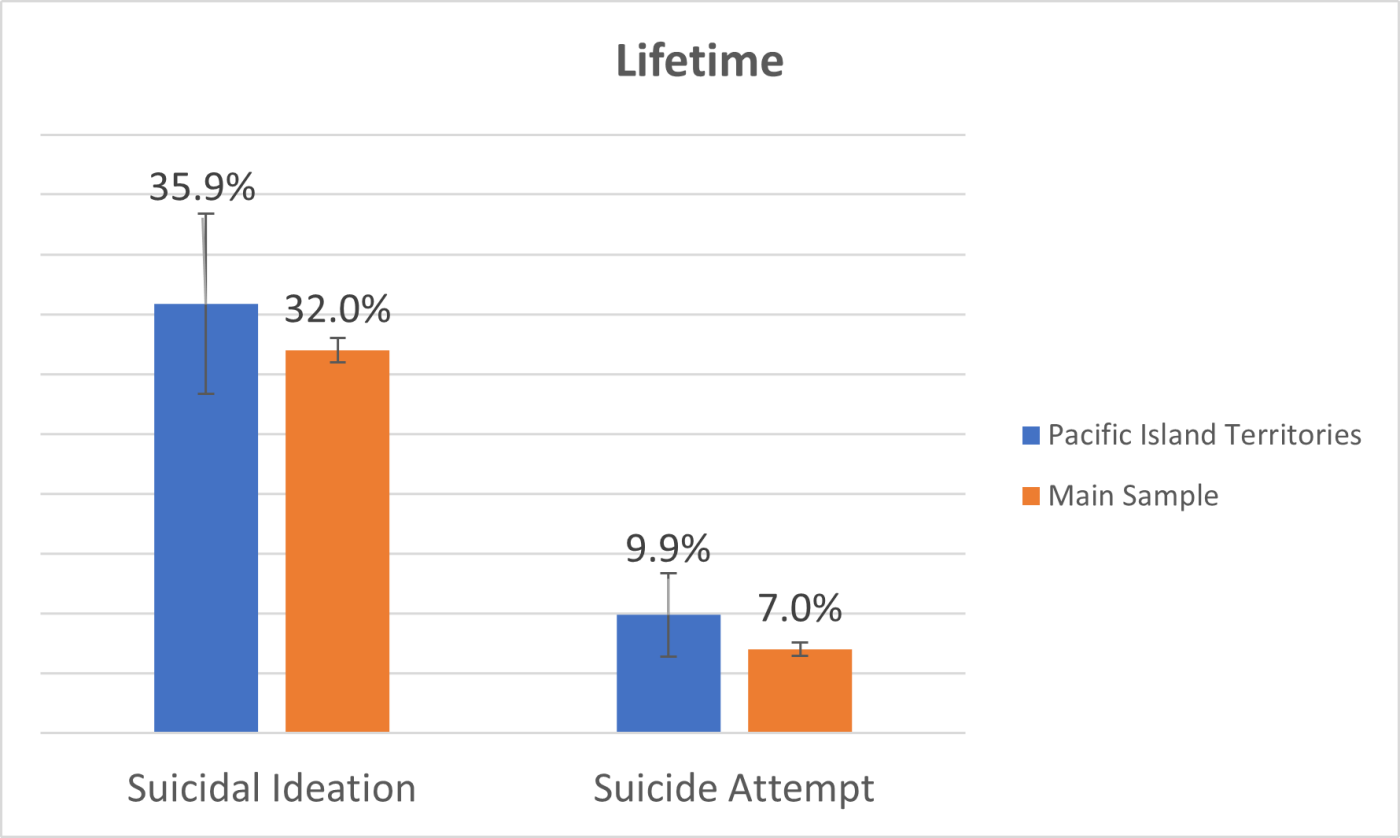
Lifetime Suicidal Ideation and Suicide Attempt Prevalence among Veterans Residing in **U.S. Pacific Island Territories, relative to Veterans in the Main ASCEND Wave 1 Sample (2022).** The main ASCEND sample included Veterans residing in all 50 **U.**S. states, DC, and Puerto Rico [28].

Lifetime SI prevalence appeared to be higher among Veterans residing in U.S. Pacific Island Territories (35.86%; 95% CI: 28.34, 43.39), compared to among Veterans in the main sample (31.98%; 95% CI: 30.97, 32.99) (Fig 2), although this difference was not statistically significant (i.e., confidence intervals overlapped). Similarly, Veterans residing in U.S. Pacific Island Territories (9.86%; 95% CI: 6.36, 13.37) had a higher prevalence of lifetime suicide attempt, relative to Veterans in the main sample (6.99%; 95% CI: 6.41–7.56), though this difference also was not statistically significant.

**Fig 3:**
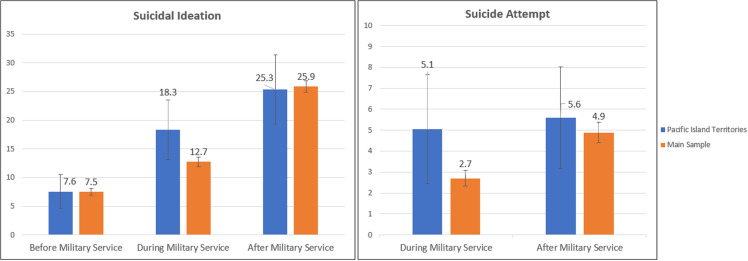
Suicidal Ideation and Non-Fatal Suicidal Self-Directed Violence Prevalence Preceding, During, and Following Military Service Among Veterans Residing in **U.S. Pacific Island Territories, relative to Veterans in the Main ASCEND Wave 1 Sample (2022)**. The main ASCEND sample included Veterans residing in all 50 U.S. states, DC, and Puerto Rico [28]. Values are not displayed for pre-military interrupted attempts and suicide attempts due to potentially unreliable estimates.

Veterans in U.S. Pacific Island Territories (18.32%; 95% CI: 13.14, 23.50) had a higher prevalence of SI during military service than Veterans in the main sample (12.72%; 95% CI: 11.90, 13.54); moreover, Veterans in U.S. Pacific Island Territories had nearly double the prevalence of suicide attempts during military service (5.05%; 95% CI: 2.46, 7.64), compared to those in the main sample (2.70%; 95% CI: 2.32, 3.07) (Fig 3). However, these differences were not statistically significant. Conversely, rates of SI preceding military service were similar between Veterans living in U.S. Pacific Island Territories (7.56%; 95% CI: 4.64, 10.47) and Veterans in the main sample (7.53%; 95% CI: 6.90, 8.15). Similarities were also observed in rates of SI following military service between Veterans in U.S. Pacific Island Territories (25.34%; 95% CI: 19.29, 31.39) and in the main sample (25.88%; 95% CI: 24.91, 26.85), as well as in rates of suicide attempts after military service between Veterans residing in U.S. Pacific Island Territories (5.60%; 95% CI: 3.17, 8.03) and those in the main sample (4.88%; 95% CI: 4.39, 5.36).

### Associations with suicidal ideation (Aim 3)

#### Pacific Island Territories.

Finally, we examined if demographic variables and VA characteristics were associated with post-military and past-year SI among Veterans residing in U.S. Pacific Island Territories (Table 6). Age was significantly associated with both post-military and past-year SI. Compared to Veterans ages 18–34, the prevalence of post-military SI was significantly lower among Veterans ages 55–74 (PR = 0.34; 95% CI: 0.19, 0.63) and 75 and older (PR = 0.29; 95% CI: 0.14, 0.62). For past-year SI, Veterans ages 18–34 had a significantly higher prevalence compared to all other age groups, ranging from 58.7% (past-year SI) lower for those ages 35–54 to 86.5% (past-year SI) lower for those ages 75 + . AI/AN Veterans had more than double the prevalence of post-military SI, compared to those who did not identify as AI/AN (PR = 2.10; 95% CI: 1.00, 4.38). Prevalence of past-year SI was also elevated among AI/AN Veterans, though this difference was not statistically significant (PR = 1.78; 95% CI: 0.50, 6.35). Veterans who identified as Samoan had a significantly lower prevalence of both post-military SI (PR = 0.19; 95% CI: 0.04, 0.89) and past-year SI (PR = 0.16; 95% CI: 0.03, 0.84), compared to those who did not identify as Samoan. Significant differences were not detected in the prevalence of post-military or past-year SI with regard to region, multi-racial identity, gender, VA service connection, enrollment in VHA care, or use of VHA services. However, the magnitude of difference in post-military SI was notable with respect to region, despite not being statistically significant; specifically, Veterans residing in CNMI had a higher prevalence of post-military and past-year SI, whereas those residing in American Samoa had a lower prevalence of post-military SI and past-year SI, relative to Veterans residing in Guam. Additionally, although not statistically significant, Veterans enrolled in VHA care had an elevated prevalence of both post-military SI (PR = 1.69; 95% CI: 0.95, 3.02) and past-year SI (PR = 1.88; 95% CI: 0.90, 3.91).

**Table 6:** Associations with post-military and past-year suicidal ideation among veterans in U.S. Pacific Island territories.

	Post-Military Suicidal Ideation								Past-Year Suicidal Ideation							
	All Pacific Island Territories (n = 533)				Guam (n = 485)				All Pacific Island Territories (n = 533)				Guam (n = 485)			
	PR	95% CI		p	PR	95% CI		p	PR	95% CI		p	PR	95% CI		p
**Age (ref = 18–34)**																
35-54	0.70	0.44	1.12	.138	0.73	0.42	1.27	.267	**0.41**	**0.024**	**0.072**	**.002**	**0.35**	**0.19**	**0.63**	**.001**
55-74	**0.34**	**0.19**	**0.63**	**.001**	**0.35**	**0.18**	**0.69**	**.003**	**0.17**	**0.09**	**0.35**	**<.001**	**0.13**	**0.06**	**0.28**	**<.001**
75 and above	**0.29**	**0.14**	**0.62**	**.001**	**0.25**	**0.11**	**0.59**	**.002**	**0.14**	**0.05**	**0.39**	**<.001**	**0.10**	**0.03**	**0.33**	**<.001**
**Region (ref = Guam)**																
Northern Mariana Islands	1.59	0.91	2.76	.100					1.87	0.91	3.84	.090				
American Samoa	0.41	0.41	1.19	.101					0.54	0.18	1.61	.266				
**Race/ethnicity** ^**1**^																
Guamanian or CHamoru	1.38	0.86	2.21	.186	1.32	0.78	2.23	.310	1.22	0.67	2.24	.519	1.22	0.61	2.41	.576
White	0.88	0.45	1.72	.700	0.86	0.42	1.74	.666	1.16	0.53	2.52	.714	1.18	0.51	2.71	.703
Filipino	0.90	0.46	1.77	.755	0.94	0.46	1.91	.864	1.00	0.40	2.52	.990	1.05	0.39	2.84	.921
Other Pacific Islander	1.52	0.92	2.51	.102	**1.69**	**1.00**	**2.86**	**.0496**	1.07	0.52	2.20	.860	1.30	0.59	2.89	.516
Black or African American	0.50	0.11	2.19	.357	0.51	0.12	2.24	.371	0.63	0.13	3.08	.563	0.66	0.13	3.27	.609
Hispanic	1.17	0.68	2.00	.572	1.06	0.59	1.89	.848	1.28	0.60	2.73	.527	1.01	0.42	2.43	.981
Samoan^2^	**0.19**	**0.04**	**0.89**	**.035**					**0.16**	**0.03**	**0.84**	**.031**				
Other Asian^3^	0.32	0.06	1.61	.168	0.31	0.06	1.60	.160	0.22	0.03	1.41	.110	0.19	0.03	1.42	.106
American Indian/Alaska Native	**2.10**	**1.00**	**4.38**	**.049**	**2.42**	**1.17**	**5.00**	**.017**	1.78	0.50	6.35	.373	2.11	0.60	7.42	.243
Japanese^2^	1.59	0.66	3.82	.301					0.87	.020	3.81	.852				
**Multi-racial**	1.10	0.49	2.47	.818	1.05	0.43	2.55	.919	1.02	0.40	2.63	.966	1.23	0.45	3.38	.688
**Gender (ref = Men)**	1.25	0.75	2.09	.392	1.30	0.75	2.27	.353	1.14	0.54	2.37	.735	1.26	0.57	2.78	.573
**VA service connection**	1.30	0.81	2.08	.270	1.41	0.85	2.35	.186	1.27	0.69	2.33	.437	1.49	0.76	2.91	.250
**Enrolled in VHA**	1.69	0.95	3.02	.075	1.82	0.96	3.42	.066	1.88	0.90	3.91	.091	2.22	0.98	5.02	.055
**Use of VHA services (ref = no use)**																
Yes, prior to the past year	1.33	0.81	2.18	.254	1.49	0.87	2.54	.143	1.05	0.55	1.98	.887	1.27	0.62	2.59	.512
Yes, in the past year	1.16	0.55	2.43	.701	1.25	0.56	2.78	.586	0.80	0.27	2.35	.685	0.91	0.28	2.99	.875

CI = confidence interval; PR = prevalence ratio. Values significant at *p* < .05 are bolded.

^1^Not mutually exclusive. For race, due to lack of mutual exclusivity between variables, the prevalence ratio was computed for each race relative to all other races, rather than using a constant reference group.

^2^Estimates for Guam not calculated due to sample *n* < 10.

^3^Included Korean, Asian Indian, Vietnamese, Chinese, and other Asian. These were included as part of a broader category due to small n’s.

#### Guam.

In the analysis specific to Veterans residing in Guam (Table 6), results were largely similar, with age and AI/AN race also significantly associated with SI among Veterans residing in Guam. In addition, Veterans in Guam who identified as “Other Pacific Islander” had a significantly higher prevalence of post-military SI (PR = 1.69; 95% CI: 1.00, 2.86).

## Discussion

Despite the significant military presence in Guam [36] and high numbers of Veterans residing in U.S. Pacific Island Territories [5–7], there has been an absence of information regarding the prevalence of SI and SSDV among Veterans in this region. In particular, while comprehensive data have been available regarding suicide rates in the general U.S. population and among U.S. Veterans for decades [1], suicide mortality data specific to U.S. Pacific Island Territories was not included in National Death Index records until 2017, and even those records likely are not comprehensive of all years [8]. Considering the absence of information regarding suicide among Veterans residing in U.S. Pacific Island Territories, the present study fills an important gap, by examining the feasibility of surveying Veterans in U.S. Pacific Island Territories on SI and NF-SSDV and by identifying the prevalence of SI and NF-SSDV among Veterans in this region, based on survey data collected in 2022.

### Feasibility

We obtained an AAPOR response rate of 21.6%, which was higher than that observed in the main ASCEND Wave 1 study (19.2% [28]), far exceeding our conservative planning estimate of 5%. The response rate obtained was despite some differences in study methods (e.g., inability to update contact information proximal to recruitment in the Pacific Islands pilot, time zone differences that could limit the utility of the telephone response option) which could have resulted in a lower response rate. These findings suggest that even though Veterans in U.S. Pacific Island Territories have often been excluded from survey-based research, including them in such research is feasible. Researchers are strongly encouraged to consider including Veterans from U.S. Pacific Island Territories in future surveys to ensure that results reflect *all* U.S. Veterans, regardless of their region of residence. Nonetheless, specific groups of Veterans residing in U.S. Pacific Island Territories responded at lower rates – namely, those whose race/ethnicity were “unknown” in broader VA datasets, those aged 18–34 years, individuals who separated from the military more recently [≤5 years prior], individuals residing in American Samoa, females, those whose race/ethnicity was coded as AANHPI, and those who had not recently used any VHA services. Addressing barriers to participating in survey research among these Veteran groups will be critical for future research focused on Veterans in U.S. Pacific Island Territories. Additionally, understanding reasons for participating in survey research among Veterans in U.S. Pacific Island Territories may help with bolstering response rates in subsequent research.

### Prevalence

Our findings also suggest a high prevalence of lifetime SI (35.86%), preparatory behaviors (11.84%), interrupted attempts (11.96%), and suicide attempts (9.86%) among Veterans residing in U.S. Pacific Island Territories. Veterans in this region appeared to have a higher prevalence of SI and suicide attempts overall (i.e., lifetime) and during their military service, relative to Veterans in the overall Veteran population, the majority of whom live in the Continental U.S., although these differences were not statistically significant. Additionally, across SI and interrupted attempts, prevalence was highest after one’s military service had concluded. These findings highlight the importance of routine suicide risk screening for Veterans in this region. Additionally, extant research underscores the need to address regional and cultural factors that may prevent communicating suicidality (e.g., stigma, specific idioms of distress) during suicide screening and risk assessment [20,37–39]).

### Suicide methods

To date, information regarding suicide methods among Veterans in U.S. Pacific Island Territories has also been limited. While sample size limitations precluded our ability to present reliable estimates for methods used in prior SSDV (i.e., suicide attempts) among Veterans in this region, as well as for all methods considered in past-year SI, we were able to report on the most commonly considered methods among Veterans with past-year SI. Consistent with findings in the overall Veteran population [28], the most common methods considered during past-year SI among Veterans in U.S. Pacific Island Territories were motor vehicle crash, overdose/poisoning, and gunshot. It is worth noting, however, that the percentage of Veterans who considered gunshot as a suicide method during past-year SI appears to be lower in Pacific Island Territories (24.30%; 95% CI: 11.98, 36.62), compared to among Veterans overall (39.60%; 95% CI: 36.17, 43.04) [28]. Considering the high case fatality rates associated with firearms [40,41], this finding has important implications. Subsequent research is warranted to determine if this finding also extends to suicide attempts and suicide deaths. Future research to identify specific methods that Veterans in each Pacific Island Territory are using in both non-fatal and fatal SSDV is necessary. As other studies have identified hanging as a common method of suicide attempts and suicide deaths in the Pacific Islands [11,15,42], it will also be important to determine the extent to which Veterans in this region consider hanging as a suicide method during experiences of SI.

### Factors associated with SI

Multiple factors were associated with SI among Veterans in the U.S. Pacific Island Territories. In particular, age was significantly associated with both post-military and past-year SI. Our findings add to a growing literature underscoring younger Veterans (those 18–34 years of age) as a particularly high-risk group, with respect to both suicide mortality [1] and post-military and past-year SI. Younger Veterans had a higher prevalence of past-year and post-military SI, relative to Veterans in older age groups. This difference was particularly stark when compared to the 75 + age group, who had 86.5% lower prevalence of past-year SI and 70.6% lower prevalence of post-military SI. Veterans ages 18–34 have the highest rates of suicide in the Veteran population [1], with AANHPI Veterans in this age group appearing to be at further elevated risk [43]. Relatedly, in Guam (though not specific to Veterans), almost half (47%) of suicide deaths between 2010–2021 were among those less than 30 years of age, and those ages 30–39 had the highest suicide rates from 2019–2021 [11]. Unfortunately, yield in the present study was lowest among younger Veterans (12.73%), suggesting that these Veterans may be harder to engage in research. To address SSDV among younger Veterans in Pacific Island Territories, it will be crucial to understand their perspectives, facilitators, and barriers to participating in NF-SSDV research, as well as effective strategies for engaging them in research.

In addition to age, race was associated with SI. Veterans who identified as AI/AN comprised 2.86% of the population of Veterans in U.S. Pacific Island Territories and 2.96% of the Veteran population in Guam. Veterans who identified as AI/AN had more than twice the prevalence of post-military SI, compared to Veterans who did not identify as AI/AN. AI/AN Veterans nationally also have high rates of SI [44] and suicide mortality [1,45] and experienced a sharp increase in suicide rates from 2020 to 2021 [1]. Nonetheless, research on AI/AN Veterans typically has not focused on U.S. Pacific Island Territories, with some reports excluding these territories [46]. Our findings underscore the importance of continued suicide prevention efforts for AI/AN Veterans [1], including among those residing in U.S. Pacific Island Territories.

Veterans in Guam who identified as “Other Pacific Islander”—who comprised 7.67% of Veterans in Guam—also had a significantly higher prevalence of post-military SI. A similar trend was observed for Veterans who identified as “Other Pacific Islander” across all Pacific Island Territories, although this was not statistically significant. In the general population of Guam, those who identified as Chuukese had the highest suicide rate in 2021 (50.4 per 100,000), relative to all other racial/ethnic groups [11]; additionally, suicide rates are high in specific Pacific Island regions, such as the Federated States of Micronesia and Kiribati [14], where individuals who have served in the U.S military are presently unable to access VA healthcare. Unfortunately, additional information regarding race and ethnicity was not collected from Veterans who identified as “Other Pacific Islander” in the present study, underscoring the need for more fine-grained assessment of race and ethnicity among these Veterans in future surveillance efforts, in line with a broader call to disaggregate different Pacific Islander groups in research [47–49].

Veterans who identified as Samoan—who comprised 12.88% of Veterans residing in U.S. Pacific Island Territories—had a lower prevalence of both post-military and past-year SI. Relatedly, Veterans who resided in American Samoa appeared to have a lower prevalence of post-military and past-year SI, whereas Veterans in CNMI appeared to have a higher prevalence of post-military and past-year SI, relative to Veterans in Guam, although these differences were not statistically significant. These findings suggest that there may be regional differences that impact the prevalence of SI among Veterans residing in U.S. Pacific Island Territories. However, there is limited scientific knowledge regarding suicide, suicide attempts, and SI in American Samoa and CNMI [15], both among Veterans and in the general population. As such, future SSDV surveillance and research in both American Samoa and CNMI is requisite and could also examine the intersection of region and culture in NF-SSDV risk.

Finally, Veterans enrolled in VHA care—57.63% of the population of Veterans in Pacific Island Territories—had a higher prevalence of post-military and past-year SI than those not enrolled in VHA care, although this difference was not statistically significant. Of note, Veterans who are enrolled in VHA services may have more health concerns that increase their risk for experiencing SI. Given the non-significant nature of this finding, future research is warranted to explore this further.

### Implications for prevention and future research

Our findings underscore the importance of SSDV surveillance efforts and suicide prevention initiatives for Veterans residing in U.S. Pacific Island Territories. Multiple community-based suicide prevention efforts are underway in different U.S. Pacific Island Territories, including the Governor’s Challenge, Together With Veterans, and community partnerships [50]. The current study provides new information that can be built upon to track changes in regional rates of SI and NF-SSDV over time, as public health-oriented suicide prevention initiatives continue. These findings can also be used in conjunction with regional public health campaigns to increase awareness of the prevalence of SI and suicide attempts among Veterans in U.S. Pacific Island Territories; if paired with culturally responsive messaging [51] and information about how Veterans can access help in these areas [52], this may increase the extent to which Veterans in Guam, CNMI, and American Samoa obtain services for SI before SSDV occurs. Moreover, increasing public awareness of the prevalence of SI and NF-SSDV among Veterans in this region, particularly following military separation, may prompt community members, family and friends, to initiate discussions about mental health and suicide and to help facilitate access to mental health care when there are concerns.

The high rates of lifetime SI and suicide attempts among Veterans in U.S. Pacific Island Territories underscore the importance of access to evidence-based mental health care, including suicide prevention, for Veterans in this region. Mental health treatment to address suicidal thoughts and to prevent suicide attempts may be hindered by cultural stigma regarding mental health concerns and suicide [20,37–39], as well as structural barriers, such as difficulty hiring healthcare providers and more limited on-island specialty and inpatient mental health services [7,20,53]; indeed, recruitment and retention of licensed healthcare providers can be challenging due to myriad factors, such as the high cost of living [20,52]. Moreover, Guam, CNMI, and American Samoa are distant from VA medical centers in Hawaii and the mainland U.S. Considering these factors, innovative health service delivery approaches (e.g., telehealth) are likely particularly important. However, these approaches only work for services that can be delivered remotely (e.g., outpatient mental health) and do not necessarily apply for more intensive treatments necessary for stabilization during an acute suicidal crisis (e.g., emergency services). As such, understanding how to facilitate prompt and easy access to both upstream and acute suicide prevention services for Veterans in U.S. Pacific Island Territories is essential.

Finally, these findings highlight necessary next steps for research to prevent suicide among Veterans in this region. In particular, research is warranted to establish whether SI and suicide attempt prevalence are indeed higher among Veterans residing in U.S. Pacific Island Territories, as well as to understand factors that may explain this. AANHPI service members and Veterans have described being misunderstood by other service members and experiencing racial profiling, discrimination, and hazing during their military service—noting these to be experiences that impacted their career advancement, quality of life, and which led to cultural isolation and mental health concerns [7]. In addition, AANHPI Veterans in Guam have described experiencing challenges obtaining employment and accessing benefits and services following their military service [53]. Racial discrimination, unemployment, and financial problems have been associated with SI and suicide attempts in other samples [54,55]. Work to identify drivers of SI and NF-SSDV among Veterans in this region and to determine optimal ways to address these factors is necessary.

### Limitations

Limitations should be noted. First, although weighted analyses accounted for potential non-response bias, the non-response analysis was conducted on all ASCEND Wave 1 respondents (i.e., combined main sample and Pacific Island Territory pilot sample) [28], and our findings suggest that non-response patterns differed somewhat in the pilot sample. Additionally, our findings assume forthright responding regarding SI and NF-SSDV. However, research suggests that the stigma regarding suicide may be heightened among individuals residing in Pacific Island Territories [20,37–39,51,53]; as such, it is possible that our estimates of SI and NF-SSDV prevalence are conservative (i.e., underestimates) due to the potential for response bias. Third, we were unable to provide comprehensive prevalence estimates specific to CNMI and American Samoa due to small cell sizes that precluded precise territory-specific estimates for these regions. Nonetheless, these locations are separate territories/commonwealths, each with different cultures, resources, and beliefs, and warrant additional research. Fourth, small sizes for some cells limited precision of estimates, which was further limiting for Guam. Relatedly, although we conducted analyses to examine the associations of different variables with SI, none of these analyses adjusted for covariates given sample sizes, and we were only able to examine SI, rather than suicide attempts. Finally, direct comparison to the main ASCEND sample was precluded due to differential sampling designs; as such, we were not able to assess whether differences in Veteran characteristics (e.g., age or sex distribution) explain the potential differences in SI and suicide attempt prevalence observed between regions.

## Conclusions

To our knowledge, this pilot study is the first study to examine SI and NF-SSDV among Veterans residing in U.S. Pacific Island Territories. The higher response rate among Veterans in this region, relative to among Veterans in the broader national sample [28], suggests that surveying Veterans in this region on SI and NF-SSDV is feasible. In addition, including these Veterans in future NF-SSDV surveillance, suicide prevention initiatives, and research is essential, considering the high rates of SI and SA, as well as the limited data available regarding suicide mortality among Veterans in this region. Our findings also suggest that there may be differences in suicide methods considered among Veterans in this region, such as a lower percentage of Veterans considering gunshot; future research to explore this further and to apply this information to method-specific suicide prevention efforts is warranted. Results from this study also highlight Veteran groups that may warrant particular attention in regional suicide prevention efforts, such as those ages 18–34 and who identify as AI/AN. Finally, considering that culturally-adapted interventions are associated with increased therapeutic benefits [56], these findings can be used to inform future research on culturally-adapted strategies to prevent suicide among Veterans residing in U.S. Pacific Island Territories.
